# Eye Care Behaviors among Adults in Poland: A Nationwide Cross-Sectional Survey

**DOI:** 10.3390/ijerph20043590

**Published:** 2023-02-17

**Authors:** Agnieszka Kamińska, Jarosław Pinkas, Piotr Tyszko, Iwona Wrześniewska-Wal, Mateusz Jankowski

**Affiliations:** 1 Faculty of Medicine, Collegium Medicum, Cardinal Stefan Wyszynski University, 01-938 Warsaw, Poland; 2 School of Public Health, Centre of Postgraduate Medical Education, 01-826 Warsaw, Poland; 3 Department of Social Medicine and Public Health, Medical University of Warsaw, 02-007 Warsaw, Poland; 4 Institute of Rural Health in Lublin, 20-090 Lublin, Poland

**Keywords:** vision impairment, eye diseases, prevention, visual hygiene, ophthalmology, Poland

## Abstract

Implementation of eye care behaviors may reduce the risk of eye symptoms and diseases. This study aimed to assess eye care behaviors and identify factors associated with eye care practices among adults in Poland. This cross-sectional survey was carried out between 9 and 12 December 2022 on a nationwide random quota sample of adults in Poland. The study questionnaire included a set of questions on 10 different eye care behaviors. The study population included 1076 participants with a mean age of 45.7 ± 16.2 years, and 54.2% of participants were females. The most common (30.2%) eye care behavior was the use of good lighting indoors, and 27.3% used sunglasses with a UV filter. More than one-fifth of participants declared taking regular screen breaks and limiting screen time. Less than one-tenth of participants used dietary supplements with lutein, beta-carotene, or zinc. Out of 12 factors assessed in this study, self-reported knowledge of eye diseases was the most important factor associated (*p* < 0.05) with eye care behaviors. There were no economic or educational gaps (*p* > 0.05) in the implementation of most of the eye care behaviors among adults in Poland. This study revealed a low level of implementation of eye care behaviors among adults in Poland.

## 1. Introduction

Approximately 2.2 billion people globally have a vision impairment [1]. Of those, more than 50% have vision impairment or blindness from potentially preventable or correctable causes [1]. Aging, lifestyle, genetics, history of infections, and chronic health conditions (e.g., diabetes or hypertension) are the most important factors for eye diseases and vision impairment [1,2,3,4,5]. Currently, most of the major causes of vision impairment and blindness have modifiable risk factors as well as effective therapy [1,3,5]. However, findings from the World report on vision, published by the World Health Organization, showed that interventions for health promotion in the field of eye care had received less attention than those aimed at secondary prevention or treatment [1,6].

There is a wide scope of eye conditions that can be targeted effectively with preventive strategies, including myopia, ocular trauma, age-related macular degeneration, cataract, trachoma, and ocular infections [1,7,8,9]. Tobacco use and a low-quality diet are common risk factors associated with numerous diseases, including eye diseases like cataracts and age-related macular degeneration [7,8]. In addition to general recommendations for a healthy lifestyle, there are also lifestyle behaviors aimed at eye care [1,9].

There is a strong correlation between ultraviolet (UV) radiation exposure and eye diseases [10,11,12]. Chronic UV light exposure to the eye has been associated with cataract formation, pterygium, climatic droplet keratopathy, and eyelid malignancies [10,11]. Wearing sunglasses with a UV filter on a sunny day is an example of eye care behavior that provides UV eye protection [12].

It is estimated that by 2050, nearly half of the world’s population may be myopic [13]. Lifestyle behaviors such as near work, incorrect reading and writing posture, increased time studying and reading, limited time outdoors, and prolonged use of electronic devices (especially mobile devices like smartphones) may lead to visual fatigue and refractive errors (mostly myopia among children) [14,15,16]. Increased time spent outdoors and decreased near-work activities are one of the most effective interventions to delay the onset and slow the progression of myopia [1,17].

The workplace also plays an important role in eye care promotion [18]. Visual ergonomics in the workplace may reduce the risk of computer vision syndrome, dry eye, and eye strain [19]. Taking regular screen breaks may also reduce the risk of eye symptoms. Moreover, proper organization of the workplace and compliance with workplace eye safety guidelines reduces the risk of work-related eye injuries [20].

Individuals with eye conditions should pay particular attention to eye care behaviors. Compliance with spectacle wear is the basic principle of eye care among individuals with refractive errors [1]. Moreover, the use of lubricating eye drops is the most available means of alleviating symptoms of dry eye [21]. Hand hygiene prior to contact lens handling also reduces the risk of common eye infections [22].

Most of the studies on eye care behaviors are limited to preventative lifestyle changes that can reduce the risk of myopia or slow the progression of myopia among children [1]. In the *World report on vision*, the WHO called on member states to provide high-quality, representative data on eye care [1]. Data on eye care behaviors among adults may be used by public health authorities and healthcare workers to develop eye care strategies both at the national and local (e.g., in the workplace) levels. There is a lack of nationwide data on eye care behaviors among adults in Poland.

Therefore, this study aimed to assess eye care behaviors among adults in Poland and to identify factors associated with eye care practices among adults in Poland.

## 2. Materials and Methods

### 2.1. Study Design

This cross-sectional survey was carried out between 9 and 12 December 2022. Data were collected using computer-assisted web interview (CAWI) methods. The study questionnaire was available online at a dedicated research platform managed by the survey company [23]. Datasets generated during the data collection process were anonymous. Participation in this study was voluntary, and informed consent was collected. The study protocol was approved by the Ethical Committee at the Centre of Postgraduate Medical Education (Warsaw, Poland), approval number: 154/2022.

### 2.2. Population

A representative sample of 1076 adults was selected from >100,000 individuals registered in the dataset managed by the survey company [23]. Non-probability quota sampling technique was applied. A nationwide representative sample was selected, with the following variables included in the stratification model: gender, age, and place of residence. Data from the public demographic registry managed by Statistics Poland were used for sampling and sample size calculations [24]. The sample size was representative of the adult population of Poland [25,26].

### 2.3. Questionnaire and Measures

This study was carried out as a part of the research project entitled “Poles’ attitudes towards eye diseases-knowledge about eye diseases, awareness of risk factors, prevention”. The study questionnaire included 9 questions (5 multiple-choice, 4 single-choice) on eye care, awareness of eye disorders and diseases, and eye care behaviors. Moreover, a set of questions on socioeconomic characteristics was addressed. The questionnaire was self-prepared based on the literature review [1,2,3,4,5,9]. A pilot study was carried out, and 11 adult subjects filled the questionnaire twice, 7 days apart. After the pilot study, two questions were revised to improve the clarity of the text.

Eye care behaviors: Participants were asked about eye care behaviors using the following question: “Which of the following eye protection and eye care behaviors have you used in the last 12 months?: (1) wearing sunglasses with a UV filter on a sunny day; (2) regular use of lubricating eye drops; (3) taking food or a dietary supplement that contains lutein; (4) taking food or a dietary supplement that contains beta-carotene; (5) taking food or a dietary supplement that contains zinc; (6) limiting the time spent on the computer; (7) taking regular breaks while working on the computer; (8) limiting the time spent on watching TV, tablet or smartphone screen; (9) use good lighting indoors, especially when reading a book; (10) eye exercises (e.g., relaxation) (yes/no)”.

Self-reported knowledge of eye diseases: Participants were asked to rate their level of knowledge of eye diseases using a 5-point Likert scale (very good, rather good, moderate, rather bad, and very bad). In the analysis, the responses very bad and rather bad were combined into one answer: “bad”, and responses “rather good” and “very good” were combined into one answer: “good”.

Socioeconomic variables: Currently employed or self-employed participants were classified as professionally active. Unemployed, retired, or students were classified as those with passive professional status. Economic status was assessed based on self-reported declaration (good, moderate, and bad financial situation of the household).

The presence of chronic diseases, as well as wearing spectacles or contact lenses, was based on self-reported declarations and medical records were not verified, as this study was anonymous.

### 2.4. Statistical Analysis

The IBM SPSS package version 28 was used for the statistical analysis. Descriptive statistics (frequencies and proportions) were used to present the distribution of categorical variables. Cross-tabulation with a chi-squared test was used for bivariate analyses. Multivariable logistic regression analyses were carried out to identify factors associated with eye care practices among adults in Poland. The use of eye care practices (10 separate analyses) was defined as the dependent variable. Twelve socioeconomic variables were included in the model (independent variables). The strength of association was presented by the odds ratio (OR) with 95% confidence intervals (95% CI). The criterion of statistical significance was set at *p* < 0.05.

## 3. Results

### 3.1. Study Population

The study population included 1076 participants, with a mean age of 45.7 ± 16.2 years, and 54.2% of participants were females (Table 1). Over 40% of participants had at least one chronic condition, and 55.6% of participants declared that they wore spectacles or contact lenses. Characteristics of the study population are presented in Table 1.

### 3.2. Eye Care Behaviors among Adults in Poland

The most common eye care behavior was the use of good lighting indoors (30.2%). Over one-quarter of participants (27.3%) used sunglasses with a UV filter on a sunny day (Table 2). More than one-fifth of participants declared taking regular breaks while working on the computer (22%), limiting the time spent on the computer (21.7%), or limiting the time spent watching TV/tablet/smartphone (21.2%). The use of lubricating eye drops was declared by 19.5% of participants. Less than one-tenth of participants used dietary supplementation of lutein (8%), beta-carotene (7.7%), or zinc (8.7%). Every third participant declared that in the last 12 months, they had not used any eye care practices. The prevalence of the use of eye care practices differed by socioeconomic factors (Table 2). In general, participants who declared a good level of knowledge of eye diseases, as well as those who wore spectacles or contact lenses, often declared eye care practices (Table 2). There were no differences (*p* > 0.05) in the prevalence of eye care practices (except the use of good lighting indoors) by occupational or economic status (Table 2). Females, compared to males, more often declared the use of sunglasses, lubricating eye drops, and good lighting indoors (*p* < 0.05). Older adults more often declared the use of sunglasses, lubricating eye drops, good lighting indoors, eye exercise practice, and taking lutein supplementation (*p* < 0.05). Details are presented in Table 2.

### 3.3. Factors Associated with the Prevention of Eye Disorders and Diseases among Adults in Poland

Table 3 shows the result of the multivariable logistic regression analysis. The level of self-reported knowledge of eye diseases was the most important factor associated (*p* < 0.05) with the prevention of eye disorders and diseases among adults in Poland (Table 3). Females were more likely to use sunglasses with a UV filter (OR: 2.39, 95% CI: 1.77–3.23, *p* < 0.001), use lubricating eye drops (OR: 1.51, 95% CI: 1.09–2.11, *p* = 0.01), and use of good lighting indoors (OR: 1.41, 95% CI: 1.06–1.87, *p* = 0.02). Participants aged 65 years and over were more likely to use sunglasses with a UV filter (OR: 2.37, 95% CI: 1.37–4.10, *p* = 0.002), use lubricating eye drops (OR: 3.05, 95% CI: 1.66–5.61, *p* < 0.001), use good lighting indoors (OR: 1.96, 95% CI: 1.16–3.32, *p* = 0.01), and use lutein supplementation (OR: 2.88, 95% CI: 1.16–7.20, *p* = 0.02). Moreover, compared to the younger group, participants aged 50–64 years were more likely to take foods or dietary supplements that contain lutein (OR: 2.48, 95% CI: 1.19–5.20, *p* = 0.02) or beta-carotene (OR: 2.14, 95% CI: 1.06–4.32, *p* = 0.03). Participants aged 34 years and over were less likely to perform eye exercises (*p* < 0.05). Those with higher education were more likely to use sunglasses with a UV filter (OR: 1.48, 95% CI: 1.10–1.99, *p* = 0.01), limit the time spent on the computer (OR: 1.47, 95% CI: 1.08–2.00, *p* = 0.02) and use good lighting indoors (OR: 1.79, 95% CI: 1.34–2.38, *p* < 0.001). Participants who lived in cities below 500,000 inhabitants were more likely to use sunglasses with a UV filter compared to those who lived in cities above 500,00 inhabitants (*p* < 0.05). Those who had children were less likely (*p* < 0.05) to use sunglasses, lubricating eyedrops, or lutein supplements (Table 3). Currently employed/self-employed participants were more likely to take regular breaks while working on the computer (OR: 1.46, 95% CI: 1.01–2.12, *p* = 0.04) and zinc supplementation (OR: 1.99, 95% CI: 1.13–3.50, *p* = 0.02). Participants with good economic status were more likely to use sunglasses with UV filter (OR: 1.52, 95% CI: 1.04–2.24, *p* = 0.03) and use good lighting indoors (OR: 1.86, 95% CI: 1.27–2.71, *p* = 0.001). Participants with chronic diseases were more likely to use sunglasses with UV filter (OR: 1.44, 95% CI: 1.07–1.94, *p* = 0.02) and lubricating eye drops (OR:1.68, 95% CI: 1.20–2.34, *p* = 0.002). Those participants who wore spectacles or contact lenses were more likely to use sunglasses with a UV filter on a sunny day (OR: 1.37, 95% CI: 1.01–1.85, *p* = 0.04), lutein supplements (OR: 2.33, 95% CI: 1.36–4.01, *p* = 0.002), and limiting time spent on the computer (OR: 1.64, 95% CI: 1.19–2.27, *p* = 0.003). Details are presented in Table 3.

## 4. Discussion

To the best of our knowledge, this is the first nationwide study on eye care behaviors among adults in Poland. Out of ten different eye care behaviors analyzed in this study, the use of good lighting indoors and UV protection with sunglasses were the most common eye care behaviors. However, one-third of adults in Poland have not implemented any eye care behaviors. Self-reported knowledge of eye diseases was the most important factor associated (*p* < 0.05) with eye care behaviors. There were no economic or educational gaps in the implementation of most of the eye care behaviors among adults in Poland.

Eye diseases and vision impairment pose significant public health challenges due to their social and economic consequences [27]. However, the WHO report on visions revealed that eye health is often missed in national health strategies [1]. Most of the public health interventions related to eye health are focused on disease diagnosis and management (e.g., providing access to ophthalmologic surgery) [1,6]. The global burden of eye diseases and vision impairment will increase due to global demographic changes related to population aging in developed countries as well as widespread access to the internet and an increase in smart device screen time in developing countries [1,2,3]. Self-care practices, including eye care behaviors, may alleviate the consequences of demographic and lifestyle changes and their implications for eye health at the individual and populational levels.

Poland is a former Eastern Bloc country with a rapid economic increase in the past two decades. Moreover, population decline (from 38 million to 34 million within the next 30 years) and population aging are expected [24]. The widespread implementation of eye care behaviors may reduce the health burden of eye diseases and control the utilization of eye care services [28]. In line with the evidence-based public health strategy, the identification of the current state of eye health behaviors may pose a basis for further eye health interventions.

Out of twelve different factors analyzed in this study, the self-reported level of knowledge of eye diseases was the most important factor associated with all analyzed eye care behaviors. Eye health literacy seems to be the most important factor that may influence eye care behaviors. Eye health promotion and education should primarily focus on strengthening individual health competencies related to eye care.

Findings from this study revealed that using good lighting indoors, especially when reading a book, was the most common eye care behavior. Ergonomic lighting is a crucial part of visual ergonomics [29]. Before the pandemic, lighting ergonomics was mostly implemented in the workplace [29]. However, the growing popularity of remote work during the COVID-19 pandemic encouraged households to organize an ergonomic workplace [30]. Moreover, the lighting of rooms (natural or artificial) is often a priority when designing new buildings and apartments. Along with the healthy building approach, the role of lighting ergonomics will increase [31]. In this study, older adults were more likely to use good lighting. We can hypothesize that this finding may result from the fact the prevalence of eye diseases and vision impairment increases with age [2,3]. Moreover, higher education and good financial status were also associated with good lighting indoors. We can hypothesize that a better economic situation often leads to better working conditions.

Chronic UV light exposure is harmful to the eye [10,11]. In this study, only 27.3% of participants declared they wore sunglasses with a UV filter on a sunny day. Out of 12 different factors analyzed in this study, nine were significantly associated with the use of sunglasses as an eye care behavior. Sunglasses are often treated as an element of clothing, often associated with fashion and image [12]. Female gender, living in urban areas, having higher education, and having good financial status were significantly associated with the use of sunglasses, which supports the thesis that sunglasses are mostly used for fashion purposes than for health purposes. However, the presence of chronic diseases and wearing spectacles or lenses were also significantly associated with the use of sunglasses. Significant differences in sunglasses use to socioeconomic factors suggest that there is a further need for eye health education. Public health campaigns should underline the importance of sunglasses with UV filters for health purposes (protection against UV radiation) [10,11,12].

Taking screen breaks and limiting screen time are common recommendations for individuals using electronic devices [32]. In this study, over one-fifth of participants declared taking screen breaks and limiting screen time. Higher education and wearing spectacles were associated with limiting computer screen time. We can hypothesize that those with higher education mostly work in offices or workplaces where a computer is a basic tool for work. Moreover, those who wear spectacles or contact lenses may be more aware of eye health issues but also more vulnerable to computer vision syndrome. Professionally active participants were more likely to take screen breaks which may result from the occupational medicine guidelines or health promotion in the workplace. Nevertheless, the percentage of adults in Poland taking regular screen breaks or limiting screen time was relatively low. Eye exercises (e.g., relaxation) are another method of eye care, but with limited scientific evidence [33]. Eye exercises may be used as a form of eye relaxation during screen breaks. However, less than 10% of participants declared the implementation of this eye care behavior. Eye care guidelines for those who use computers, developed by public health authorities, are needed.

Lubricating eye drops provide moisture and relief for dry eyes due to temporary causes, like being tired or due to environmental factors [21,34]. Lubricating eye drops are widely available over the counter in pharmacies and drugstores [21,34]. In this study, almost one-fifth of participants declared regular use of lubricating eye drops. Females, older adults, as well as those with chronic diseases were more likely to use lubricating eye drops. This finding is in line with the epidemiology of eye symptoms and eye conditions [1,2,3]. Menopausal women are at greater risk for developing dry eye syndrome [35]. In older age, age-related anatomical changes in the lacrimal gland, conjunctiva, and meibomian gland are observed that may affect the ocular surface health and risk of dry eyes [36]. As there were no differences in the use of lubricating eye drops by wearing spectacles/contact lenses, ophthalmologists should pay more attention to educating patients with vision impairment on the role of lubricating eye drops in eye self-care.

Diet quality may influence the risk of eye diseases [37]. Moreover, there is a scientific debate on micronutrients and their effect on eye health [38,39,40]. Lutein, beta-carotene, and zinc are often marketed as substances supporting eye health [38,39]. Moreover, dietary supplements containing the abovementioned substances were widely marketed in Poland. However, scientific evidence on the effectiveness of lutein, beta-carotene, or zinc supplementation on eye health is unclear [38,39,40]. In this study, questions on dietary supplementation and eye exercises were addressed to assess the prevalence of behaviors that may result from marketing campaigns (including social media) rather than evidence-based knowledge provided by healthcare professionals. In this study, less than one-tenth of participants declared the supplementation of lutein, beta-carotene, or zinc. Participants aged 50 years and over, as well as those wearing spectacles or contact lenses, were more likely to use lutein-containing products. Dietary supplements with lutein were the most common ones, targeted at individuals with eye conditions by supplement manufacturers. Public health institutions should pay more attention to dietary guidelines for those with eye diseases and disorders.

### 4.1. Practical Implications

Detailed characteristics of eye care behaviors in a nationwide sample of adults in Poland presented in this study may pose a basis for eye care programs and eye health strategies. Scientific data are necessary to implement evidence-based public interventions aimed at eye care. As eye health was missed in the national health strategy in Poland, this study may inform policymakers about the self-prevention methods implemented by adults in Poland. Moreover, socioeconomic characteristics and factors associated with eye care behaviors presented in this study may contribute to the current state of knowledge of health inequalities in Poland. The rapid aging of the Polish population and the increase in the number of mobile device users [25] may lead to a growing burden of eye disease and vision impairment in Poland. Information on eye care behaviors may be used by healthcare workers to educate their patients on eye health.

### 4.2. Limitations

This is a cross-sectional survey, so we cannot exclude recall bias. Moreover, data were collected using computer-assisted web interviews, so only internet users participated in this study. Nevertheless, the sample size is representative of the general adult population of Poland. Knowledge of eye diseases was self-reported based on a 5-point Likert scale. Further studies should use more advanced research items to provide a more comprehensive analysis. In this study, only ten eye care behaviors were assessed. Further studies should assess eye care behaviors in different subgroups, including patients with chronic eye conditions.

## 5. Conclusions

This study revealed a low level of implementation of eye care behaviors among adults in Poland. Self-reported eye health knowledge was the most important factor associated with eye care practices, which suggests the need to strengthen eye health competencies in the general population. There were no significant economic or educational barriers in the implementation of eye care behaviors, so education on eye health may be free of socioeconomic barriers. There is an urgent need for eye health education. Public health policies should support workplace eye care programs, as well as the promotion of visual ergonomics in educational institutions.

## Figures and Tables

**Table 1:** Characteristics of the study population (*n* = 1076).

Variable	*n*	%
Gender		
male	493	45.8
female	583	54.2
Age group (years)		
18–34	337	31.3
35–49	279	25.9
50–64	298	27.7
65+	162	15.1
Educational level		
primary	24	2.2
vocational	115	10.7
secondary	494	45.9
higher	443	41.2
Currently married		
yes	561	52.1
no	515	47.9
Place of residence		
rural area	403	37.5
city < 20,000 inhabitants	136	12.6
city ≥ 20,000 < 100,000 inhabitants	212	19.7
city ≥ 100,000 < 500,000 inhabitants	191	17.8
city ≥ 500,000 inhabitants	134	12.5
Having children		
yes	688	63.9
no	388	36.1
Household size		
living alone	148	13.8
at least two persons	928	86.2
Professional activity		
active	653	60.7
passive	423	39.3
Economic status		
good	414	38.5
moderate	408	37.9
bad	254	23.6
Health status		
presence of at least one chronic condition	478	44.4
healthy	598	55.6
Wearing spectacles or contact lenses		
yes	598	55.6
no	482	44.8
Self-reported knowledge of eye diseases		
good	107	9.9
moderate	471	43.8
bad	498	46.3

**Table 2:** Eye care behaviors among adults in Poland (*n* = 1076).

	Wearing Sunglasses with a UV Filter on a Sunny Day			Regular Use of Lubricating Eye Drops			Taking Food or a Dietary Supplement That Contains Lutein			Taking Food or a Dietary Supplement That Contains Beta-Carotene			Taking Food or a Dietary Supplement That Contains Zinc		
Variable	*n*	%	*p*	*n*	%	*p*	*n*	%	*p*	*n*	%	*p*	*n*	%	*p*
Overall	294	27.3		210	19.5		86	8.0		83	7.7		94	8.7	
Gender															
male	93	18.9	<0.001	80	16.2	0.01	52	8.9	0.2	53	9.1	0.07	53	9.1	0.7
female	201	34.5		130	22.3		34	6.9		30	6.1		41	8.3	
Age group (years)															
18–34	81	24.0	0.01	60	17.8	0.02	17	5.0	0.02	25	7.4	0.6	35	10.4	0.1
35–49	64	22.9		44	15.8		19	6.8		20	7.2		19	6.8	
50–64	90	30.2		61	20.5		32	10.7		28	9.4		31	10.4	
65+	59	36.4		45	27.8		18	11.1		10	6.2		9	5.6	
Educational level															
primary	3	12.5	<0.001	2	8.3	0.1	0	0.0	0.3	3	12.5	0.2	3	12.5	0.1
vocational	16	13.9		15	13.0		7	6.1		7	6.1		7	6.1	
secondary	131	26.5		97	19.6		38	7.7		31	6.3		35	7.1	
higher	144	32.5		96	21.7		41	9.3		42	9.5		49	11.1	
Currently married															
yes	155	27.6	0.9	112	20.0	0.7	44	7.8	0.9	42	7.5	0.8	41	7.3	0.1
no	139	27.0		98	19.0		42	8.2		41	8.0		53	10.3	
Place of residence															
rural area	95	23.6	0.04	73	18.1	0.8	25	6.2	0.3	27	6.7	0.2	27	6.7	0.2
city < 20,000 inhabitants	39	28.7		27	19.9		11	8.1		6	4.4		11	8.1	
city ≥ 20,000 < 100,000 inhabitants	68	32.1		48	22.6		17	8.0		18	8.5		20	9.4	
city ≥ 100,000 < 500,000 inhabitants	62	32.5		37	19.4		22	11.5		21	11.0		24	12.6	
city ≥ 500,000 inhabitants	30	22.4		25	18.7		11	8.2		11	8.2		12	9.0	
Having children															
yes	188	27.3	0.9	124	18.0	0.1	53	7.7	0.6	48	7.0	0.2	49	7.1	0.01
no	106	27.3		86	22.2		33	8.5		35	9.0		45	11.6	
Household size															
living alone	47	31.8	0.2	27	18.2	0.8	17	11.5	0.1	10	6.8	0.6	16	10.6	0.3
at least two persons	247	26.6		183	19.7		69	7.4		73	7.9		78	8.4	
Professional activity															
active	172	26.3	0.4	123	18.8	0.5	49	7.5	0.5	54	8.3	0.4	69	10.6	0.01
passive	122	28.8		87	20.6		37	8.7		29	6.9		25	5.9	
Economic status															
good	127	30.7	0.1	86	20.8	0.4	28	6.8	0.5	36	8.7	0.2	43	10.4	0.3
moderate	106	26.0		71	17.4		36	8.8		34	8.3		31	7.6	
bad	61	24.0		53	20.9		22	8.7		13	5.1		20	7.9	
Health status															
presence of at least one chronic condition	156	32.6	<0.001	118	24.7	<0.001	46	9.6	0.08	34	7.1	0.5	48	10.0	0.2
healthy	138	23.1		92	15.4		40	6.7		49	8.2		46	7.7	
Wearing spectacles or contact lenses															
yes	191	32.2	<0.001	134	22.6	0.01	66	11.1	<0.001	47	7.9	0.8	57	9.6	0.3
no	103	21.4		76	15.8		20	4.1		36	7.5		37	7.7	
Self-reported knowledge of eye diseases															
good	39	36.4	0.004	36	33.6	<0.001	13	12.1	0.01	15	14.0	<0.001	15	14.0	0.01
moderate	141	29.9		105	22.3		46	9.8		47	10.0		49	10.4	
bad	114	22.9		69	13.9		27	5.4		21	4.2		30	6.0	
	**Limiting the Time Spent on the Computer**			**Taking Regular Breaks while Working on the Computer**			**Limiting the Time Spent on Watching TV, Tablet, or Smartphone Screen**			**Use of Good Lighting Indoors**			**Eye Exercises** **(e.g., Relaxation)**		
**Variable**	***n***	**%**	***p***	***n***	**%**	***p***	***n***	**%**	***p***	***n***	**%**	***p***	***n***	**%**	***p***
Overall	234	21.7		237	22.0		228	21.2		325	30.2		98	9.1	
Gender															
male	104	21.1	0.6	108	21.9	0.9	97	19.7	0.3	126	25.6	0.002	52	10.5	0.1
female	130	22.3		129	22.1		131	22.5		199	34.1		46	7.9	
Age group (years)															
18–34	71	21.1	0.3	68	20.2	0.2	65	19.3	0.04	87	25.8	<0.001	46	13.6	0.01
35–49	55	19.7		53	19.0		47	16.8		66	23.7		22	7.9	
50–64	64	21.5		74	24.8		75	25.2		100	33.6		20	6.7	
65+	44	27.2		42	25.9		41	25.3		72	44.4		10	6.2	
Educational level															
primary	3	12.5	0.01	2	8.3	0.02	5	20.8	0.1	4	16.7	<0.001	1	4.2	0.8
vocational	17	14.8		20	17.4		25	21.7		13	11.3		10	8.7	
secondary	98	19.8		99	20.0		90	18.2		142	28.7		44	8.9	
higher	116	26.2		116	26.2		108	24.4		166	37.5		43	9.7	
Currently married															
yes	130	23.2	0.2	115	20.5	0.2	121	21.6	0.8	169	30.1	0.9	46	8.2	0.3
no	104	20.2		122	23.7		107	20.8		156	30.3		52	10.1	
Place of residence															
rural area	95	23.6	0.1	84	20.8	0.1	82	20.3	0.8	111	27.5	0.5	29	7.2	0.04
city < 20,000 inhabitants	23	16.9		24	17.6		28	20.6		41	30.1		7	5.1	
city ≥ 20,000 < 100,000 inhabitants	55	25.9		50	23.6		52	24.5		64	30.2		23	10.8	
city ≥ 100,000 < 500,000 inhabitants	34	17.8		39	20.4		40	20.9		66	34.6		20	10.5	
city ≥ 500,000 inhabitants	27	20.1		40	29.9		26	19.4		43	32.1		19	14.2	
Having children															
yes	155	22.5	0.4	152	22.1	0.9	159	23.1	0.04	226	32.8	0.01	53	7.7	0.03
no	79	20.4		85	21.9		69	17.8		99	25.5		45	11.6	
Household size															
living alone	28	18.9	0.4	38	25.7	0.2	32	21.6	0.9	51	34.5	0.2	14	9.5	0.9
at least two persons	206	22.2		199	21.4		196	21.1		274	29.5		84	9.1	
Professional activity															
active	146	22.4	0.5	151	23.1	0.3	135	20.7	0.6	179	27.4	0.01	65	10.0	0.2
passive	88	20.8		86	20.3		93	22.0		146	34.5		33	7.8	
Economic status															
good	93	22.5	0.6	98	23.7	0.5	94	22.7	0.5	146	35.3	0.01	42	10.1	0.5
moderate	92	22.5		88	21.6		86	21.1		117	28.7		37	9.1	
bad	49	19.3		51	20.1		48	18.9		62	24.4		19	7.5	
Health status															
presence of at least one chronic condition	112	23.4	0.2	119	24.9	0.04	117	24.5	0.02	170	35.6	<0.001	39	8.2	0.3
healthy	122	20.4		118	19.7		111	18.6		155	25.9		59	9.9	
Wearing spectacles or contact lenses															
yes	154	25.9	<0.001	152	25.6	0.02	144	24.2	0.01	209	35.2	<0.001	63	10.6	0.06
no	80	16.6		85	17.6		84	17.4		116	24.1		35	7.3	
Self-reported knowledge of eye diseases															
good	27	25.2	0.001	32	29.9	<0.001	32	29.9	<0.001	42	39.3	0.001	16	15.0	<0.001
moderate	123	26.1		131	27.8		115	24.4		159	33.8		55	11.7	
bad	84	16.9		74	14.9		81	16.3		124	24.9		27	5.4	

**Table 3:** Factors associated with the use of eye care practices among adults in Poland (*n* = 1076).

	Wearing Sunglasses with a UV Filteron a Sunny Day		Regular Use of Lubricating Eye Drops		Taking Food or a Dietary Supplement That Contains Lutein		Taking Food or a Dietary Supplement That Contains Beta-Carotene		Taking Food ora Dietary Supplement That Contains Zinc	
Variable	OR (95% CI)	*p*	OR (95% CI)	*p*	OR (95% CI)	*p*	OR (95% CI)	*p.*	OR (95% CI)	*p*
Gender										
male	1.00		1.00		1.00		1.00		1.00	
female	2.39 (1.77–3.23)	<0.001	1.51 (1.09–2.11)	0.01	1.15 (0.71–1.85)	0.6	1.60 (0.98–2.61)	0.06	1.13 (0.72–1.78)	0.6
Age group (years)										
18–34	1.00		1.00		1.00		1.00		1.00	
35–49	1.06 (0.70–1.63)	0.8	1.05 (0.65–1.70)	0.8	1.69 (0.81–3.54)	0.2	1.30 (0.66–2.57)	0.5	0.77 (0.41–1.48)	0.4
50–64	1.49 (0.96–2.31)	0.07	1.50 (0.92–2.46)	0.1	2.48 (1.19–5.20)	0.02	2.14 (1.06–4.32)	0.03	1.43 (0.75–2.72)	0.3
65+	2.37 (1.37–4.10)	0.002	3.05 (1.66–5.61)	<0.001	2.88 (1.16–7.20)	0.02	1.71 (0.65–4.49)	0.3	1.00 (0.38–2.63)	0.9
Educational level										
higher	1.48 (1.10–1.99)	0.01	1.15 (0.83–1.60)	0.4	1.22 (0.76–1.96)	0.4	1.38 (0.86–2.22)	0.2	1.35 (0.86–2.12)	0.2
less than higher	1.00		1.00		1.00		1.00		1.00	
Currently married										
yes	1.13 (0.78–1.63)	0.5	1.20 (0.79–1.81)	0.4	1.11 (0.60–2.02)	0.7	1.04 (0.57–1.87)	0.9	0.85 (0.48–1.51)	0.6
no	1.00		1.00		1.00		1.00		1.00	
Place of residence										
rural area	1.61 (0.97–2.67)	0.07	1.43 (0.82–2.48)	0.2	1.08 (0.49–2.38)	0.9	1.00 (0.46–2.17)	0.9	1.09 (0.51–2.33)	0.8
city < 20,000 inhabitants	1.89 (1.05–3.40)	0.03	1.43 (0.75–2.72)	0.3	1.35 (0.54–3.36)	0.5	0.59 (0.21–1.70)	0.3	1.27 (0.52–3.09)	0.6
city ≥ 20,000 < 100,000 inhabitants	2.09 (1.23–3.57)	0.01	1.58 (0.89–2.82)	0.1	1.19 (0.52–2.71)	0.7	1.17 (0.52–2.65)	0.7	1.36 (0.62–2.97)	0.4
city ≥ 100,000 < 500,000 inhabitants	2.14 (1.25–3.67)	0.01	1.36 (0.75–2.48)	0.3	1.89 (0.85–4.21)	0.1	1.61 (0.73–3.58)	0.2	1.84 (0.86–3.93)	0.1
city ≥ 500,000 inhabitants	1.00		1.00		1.00		1.00		1.00	
Having children										
yes	0.65 (0.44–0.95)	0.03	0.44 (0.29–0.68)	<0.001	0.53 (0.29–0.96)	0.04	0.52 (0.28–0.96)	0.04	0.57 (0.32–1.02)	0.06
no	1.00		1.00		1.00		1.00		1.00	
Household size										
living alone	1.21 (0.76–1.92)	0.4	0.73 (0.43–1.26)	0.3	1.28 (0.64–2.57)	0.5	0.76 (0.35–1.66)	0.5	1.16 (0.60–2.26)	0.7
at least two persons	1.00		1.00		1.00		1.00		1.00	
Professional activity										
active	1.31 (0.93–1.86)	0.1	1.44 (0.97–2.13)	0.1	1.18 (0.67–2.07)	0.6	1.21 (0.69–2.11)	0.5	1.99 (1.13–3.50)	0.02
passive	1.00		1.00		1.00		1.00		1.00	
Economic status										
good	1.52 (1.04–2.24)	0.03	0.98 (0.65–1.49)	0.9	0.77 (0.42–1.43)	0.4	1.69 (0.85–3.36)	0.1	1.31 (0.73–2.35)	0.4
moderate	1.12 (0.76–1.64)	0.6	0.79 (0.52–1.20)	0.3	1.02 (0.57–1.81)	0.9	1.70 (0.87–3.35)	0.1	0.93 (0.51–1.71)	0.8
bad	1.00		1.00		1.00		1.00		1.00	
Health status										
presence of at least one chronic condition	1.44 (1.07–1.94)	0.02	1.68 (1.20–2.34)	0.002	1.16 (0.72–1.87)	0.5	0.83 (0.51–1.35)	0.5	1.54 (0.97–2.45)	0.07
healthy	1.00		1.00		1.00		1.00		1.00	
Wearing spectacles or contact lenses										
yes	1.37 (1.01–1.85)	0.04	1.28 (0.91–1.78)	0.2	2.33 (1.36–4.01)	0.002	0.84 (0.51–1.36)	0.5	1.11 (0.70–1.77)	0.7
no	1.00		1.00		1.00		1.00		1.00	
Self-reported knowledge of eye diseases										
good	1.85 (1.15–2.97)	0.01	3.29 (1.99–5.43)	<0.001	2.68 (1.28–5.58)	0.01	3.74 (1.81–7.72)	<0.001	2.37 (1.19–4.71)	0.01
moderate	1.30 (0.96–1.77)	0.1	1.77 (1.25–2.50)	0.001	1.74 (1.05–2.90)	0.03	2.42 (1.41–4.12)	0.001	1.88 (1.15–3.07)	0.01
bad	1.00		1.00		1.00		1.00		1.00	
	**Limiting the Time Spent on the Computer**		**Taking Regular Breaks while Working on the Computer**		**Limiting the Time Spent on Watching TV, Tablet, or Smartphone Screen**		**Use of Good Lighting Indoors**		**Eye Exercises (e.g., Relaxation)**	
**Variable**	**OR (95%CI)**	***p***	**OR (95%CI)**	***p***	**OR (95%CI)**	***p***	**OR (95%CI)**	***p***	**OR (95%CI)**	***p***
Gender										
male	1.00		1.00		1.00		1.00		1.00	
female	1.03 (0.76–1.41)	0.8	0.93 (0.68–1.26)	0.6	1.07 (0.78–1.46)	0.7	1.41 (1.06–1.87)	0.02	0.64 (0.41–1.01)	0.05
Age group (years)										
18–34	1.00		1.00		1.00		1.00		1.00	
35–49	0.82 (0.53–1.27)	0.4	0.92 (0.59–1.44)	0.7	0.74 (0.47–1.17)	0.2	0.84 (0.56–1.27)	0.4	0.51 (0.28–0.93	0.03
50–64	0.87 (0.55–1.39)	0.6	1.37 (0.86–2.17)	0.2	1.19 (0.75–1.88)	0.5	1.32 (0.87–2.02)	0.2	0.39 (0.20–0.76)	0.01
65+	1.53 (0.86–2.72)	0.2	1.74 (0.96–3.14)	0.1	1.23 (0.68–2.20)	0.5	1.96 (1.16–3.32)	0.01	0.36 (0.15–0.88)	0.03
Educational level										
higher	1.47 (1.08–2.00)	0.02	1.34 (0.98–1.83)	0.1	1.34 (0.98–1.84)	0.1	1.79 (1.34–2.38)	<0.001	0.88 (0.56–1.39)	0.6
less than higher	1.00		1.00		1.00		1.00		1.00	
Currently married										
yes	1.08 (0.73–1.60)	0.7	0.70 (0.48–1.04)	0.1	0.79 (0.53–1.17)	0.2	0.72 (0.50–1.04)	0.1	1.15 (0.64–2.05)	0.6
no	1.00		1.00		1.00		1.00		1.00	
Place of residence										
rural area	1.57 (0.94–2.62)	0.1	0.81 (0.50–1.30)	0.4	1.32 (0.78–2.22)	0.3	1.08 (0.68–1.72)	0.7	0.51 (0.26–0.99)	0.04
city < 20,000 inhabitants	0.97 (0.51–1.82)	0.9	0.61 (0.33–1.11)	0.1	1.25 (0.67–2.32)	0.5	1.11 (0.64–1.93)	0.7	0.37 (0.14–0.94)	0.04
city ≥ 20,000 < 100,000 inhabitants	1.55 (0.90–2.66)	0.1	0.79 (0.47–1.31)	0.4	1.45 (0.84–2.51)	0.2	1.00 (0.61–1.65)	0.9	0.75 (0.38–1.50)	0.4
city ≥ 100,000 < 500,000 inhabitants	0.95 (0.54–1.70)	0.9	0.67 (0.40–1.14)	0.1	1.21 (0.69–2.14)	0.5	1.28 (0.78–2.10)	0.4	0.75 (0.37–1.51)	0.4
city ≥ 500,000 inhabitants	1.00		1.00		1.00		1.00		1.00	
Having children										
yes	0.94 (0.62–1.41)	0.8	1.00 (0.60–1.57)	0.9	1.38 (0.91–2.10)	0.1	1.35 (0.92–1.97)	0.1	0.88 (0.49–1.58)	0.7
no	1.00		1.00		1.00		1.00		1.00	
Household size										
living alone	0.81 (0.49–1.36)	0.4	0.97 (0.60–1.57)	0.9	0.95 (0.57–1.56)	0.6	1.02 (0.65–1.60)	0.9	1.07 (0.54–2.14)	0.8
at least two persons	1.00		1.00		1.00		1.00		1.00	
Professional activity										
active	1.35 (0.93–1.97)	0.1	1.46 (1.01–2.12)	0.04	1.10 (0.76–1.58)	0.6	0.92 (0.66–1.28)	0.6	0.98 (0.58–1.66)	0.9
passive	1.00		1.00		1.00		1.00		1.00	
Economic status										
good	1.11 (0.73–1.67)	0.6	1.21 (0.81–1.82)	0.4	1.27 (0.84–1.92)	0.3	1.86 (1.27–2.71)	0.001	1.07 (0.59–1.94)	0.8
moderate	1.21 (0.81–1.81)	0.3	1.09 (0.73–1.63)	0.7	1.15 (0.77–1.73)	0.5	1.27 (0.87–1.84)	0.2	1.09 (0.60–1.98)	0.8
bad	1.00		1.00		1.00		1.00		1.00	
Health status										
presence of at least one chronic condition	1.15 (0.84–1.58)	0.4	1.32 (0.96–1.81)	0.1	1.32 (0.96–1.82)	0.1	1.36 (1.02–1.82)	0.04	0.92 (0.58–1.47)	0.7
healthy	1.00		1.00		1.00		1.00		1.00	
Wearing spectacles or contact lenses										
yes	1.64 (1.19–2.27)	0.003	1.34 (0.98–1.86)	0.1	1.25 (0.91–1.73)	0.2	1.28 (0.96–1.71)	0.1	1.75 (1.10–2.79)	0.02
no	1.00		1.00		1.00		1.00		1.00	
Self-reported knowledge of eye diseases										
good	1.59 (0.95–2.66)	0.1	2.31 (1.40–3.81)	0.001	2.14 (1.31–3.51)	0.003	1.80 (1.13–2.87)	0.01	2.57 (1.29–5.12)	0.01
moderate	1.68 (1.22–2.32)	0.002	2.17 (1.56–3.02)	<0.001	1.57 (1.13–2.18)	0.01	1.44 (1.08–1.94)	0.02	2.51 (1.53–4.12)	<0.001
bad	1.00		1.00		1.00		1.00		1.00	

## Data Availability

Data are available on reasonable request.
